# Research on Intrusion Detection Method Based on Transformer and CNN-BiLSTM in Internet of Things

**DOI:** 10.3390/s25092725

**Published:** 2025-04-25

**Authors:** Chunhui Zhang, Jian Li, Naile Wang, Dejun Zhang

**Affiliations:** School of Cyberspace Security, Beijing University of Post and Telecommunications, Beijing 100876, China; zhangchunhui@bupt.edu.cn (C.Z.); wangnaile@bupt.edu.cn (N.W.); zhangdejun@bupt.edu.cn (D.Z.)

**Keywords:** IoT security, intrusion detection, deep learning, data imbalance, feature selection

## Abstract

With the widespread deployment of Internet of Things (IoT) devices, their complex network environments and open communication modes have made them prime targets for cyberattacks. Traditional Intrusion Detection Systems (IDS) face challenges in handling complex attack types, data imbalance, and feature extraction difficulties in IoT environments. Accurately detecting abnormal traffic in IoT has become increasingly critical. To address the limitation of single models in comprehensively capturing the diverse features of IoT traffic, this paper proposes a hybrid model based on CNN-BiLSTM-Transformer, which better handles complex features and long-sequence dependencies in intrusion detection. To address the issue of data class imbalance, the Borderline-SMOTE method is introduced to enhance the model’s ability to recognize minority class attack samples. To tackle the problem of redundant features in the original dataset, a comprehensive feature selection strategy combining XGBoost, Chi-square (Chi2), and Mutual Information is adopted to ensure the model focuses on the most discriminative features. Experimental validation demonstrates that the proposed method achieves 99.80% accuracy on the CIC-IDS 2017 dataset and 97.95% accuracy on the BoT-IoT dataset, significantly outperforming traditional intrusion detection methods, proving its efficiency and accuracy in detecting abnormal traffic in IoT environments.

## 1. Introduction

As IoT devices rapidly evolve, their open and heterogeneous nature makes them increasingly vulnerable to cybersecurity threats. At the same time, the complexity and frequency of attacks in IoT environments continue to increase, posing significant challenges to IoT security [1]. Intrusion Detection Systems (IDS) play a critical role in preventing cyber-attacks by monitoring traffic in real time and identifying potential threats [2]. Mozi, a P2P botnet targeting IoT devices, spreads by exploiting weak passwords and known vulnerabilities to launch DDoS attacks and steal data from IoT devices. By 2021, Mozi had infected more than 1 million devices [3]. On 24 August 2024, four days after the release of Black Myth: Wukong, Steam services were disrupted by a DDoS attack, rendering the platform inaccessible to global players. Analysis by Qi An Xin XLab revealed that the attack used dozens of botnet-controlled nodes, with a total command volume of 280,000 instances [4]. Since 3 January 2025, DeepSeek Inc. has experienced large-scale malicious cyberattacks, starting with HTTP proxy attacks, escalating to SSDP/NTP reflection amplification attacks, and evolving to application layer DDoS attacks and brute force cracking of web servers by 27 January. By 30 January, the number of attack commands had increased a hundredfold, preventing legitimate users from accessing services [5]. Consequently, the development of an intrusion detection technology that can maintain a high detection rate while significantly reducing the false alarm rate has emerged as the core challenge in current research on ensuring the security of the Internet of Things.

A model proposed in [6] tackled the challenge of limited recognition capabilities in traditional intrusion detection systems when handling complex attack patterns by proposing a model that integrates CNN, BiLSTM, and an attention mechanism. Additionally, the model replaced the Softmax classifier with a C5.0 decision tree, achieving 95.50% accuracy on the KDD CUP 99 dataset. However, this model did not incorporate the Transformer architecture, thus lacking the ability to model global dependencies. In this paper, we introduce the Transformer module to this model to more effectively capture global features. An architecture combining 1D-CNN, BiLSTM, and an attention mechanism was developed in [7] to address insufficient feature extraction in IoT environments, achieving an AUC-ROC score of 0.995 on the KDD CUP 99 dataset. Although this model improved traffic feature recognition, it neglected feature selection and data imbalance issues. This paper introduces multiple feature selection strategies and the Borderline-SMOTE method to help the model focus on key features and better recognize minority class samples. To handle data imbalance, a CNN-BiLSTM model combined with SMOTE-ENN for data augmentation was proposed in [8], achieving 97.7% accuracy on CIC-IDS2017. While this effectively enhanced the detection of minority class samples, the model failed to capture global dependency information. The model described in this paper incorporates the Transformer module to strengthen the global modeling of sequence features and integrates multiple feature selection mechanisms to further improve the model’s robustness. A model based on Bayesian CNN and PCA was introduced in [9] to address the challenges of processing high-dimensional data in cloud DDoS attacks, achieving 99.66% accuracy on CIC-DDoS 2019. However, their study did not adequately model the temporal characteristics of traffic. This paper compensates for this deficiency by combining BiLSTM with Transformer to better capture temporal dependencies and contextual information. To improve IDS performance, the EvoBMF multi-objective evolutionary deep learning model was designed in [10], integrating BiLSTM, MHA, and SMOTE, achieving 98.97% accuracy on CIC-IDS2018. Despite its effectiveness, their method has a complex structure and high deployment costs. In contrast, the model presented in this paper employs lightweight feature selection and data augmentation techniques, reducing computational demands while maintaining high accuracy. To enhance multi-scale feature modeling, the IDS-MTran model was introduced in [11], which extracts deep features using multi-scale convolution and Transformer and enhances feature representation through the PwP method. However, their model did not consider temporal structure modeling. This paper addresses this limitation by introducing BiLSTM to effectively capture time-domain information. For intrusion detection in resource-constrained scenarios, a lightweight model combining 2D-CNN and BiLSTM was proposed in [12], using SMOTE-ENN to balance sample distribution and achieve a balance between detection performance and resource consumption. However, their study did not incorporate the Transformer structure, limiting its global modeling capability. This paper introduces the Transformer structure to the model while maintaining low computational complexity to deepen the model’s modeling capacity. A model combining CNN and LSTM was developed in [13] to address feature redundancy and temporal modeling challenges. Recursive feature elimination (RFE) was used to optimize the feature subset, yielding favorable results on NSL-KDD. However, the feature selection approach was simplistic and lacked consideration of global relationships. This paper integrates multiple feature selection strategies and introduces the Transformer module to enhance expressiveness in both spatial and temporal dimensions. A CNN-LSTM model was presented in [14] to extract spatial and temporal features, and it performed well on UNSW-NB15 and X-IIoTID. However, this study did not integrate an attention mechanism or global modeling capability. This paper enhances global information modeling through Transformer, enabling more effective identification of complex attack behaviors. An IDS model based on Transformer was proposed in [15], utilizing the self-attention mechanism to model complex network patterns, achieving over 98% accuracy on NSL-KDD. While it possesses global modeling capabilities, their model lacks CNN and RNN structures, making it challenging to fully leverage local and temporal features. This paper integrates CNN, BiLSTM, and Transformer to comprehensively combine three types of feature information, achieving higher detection accuracy. Regarding the reduction of reliance on manual feature extraction, a deep neural network model for automatic traffic feature extraction was proposed in [16], achieving 90.2% accuracy on NSL-KDD. Although straightforward, this method fails to address feature selection and data imbalance issues.

Based on the above literature review, current intrusion detection methods exhibit certain limitations: (1) Single models struggle to comprehensively capture various characteristics of network traffic (e.g., [9,11,15], etc.). (2) The issue of data category imbalance remains inadequately addressed (e.g., [7,11], etc.), causing models to favor the prediction of dominant categories during training, thereby affecting overall detection performance. (3) Many methods fail to conduct effective feature selection (e.g., [6,13], etc.), leading to too many redundant features in the original dataset.

To deal with these challenges, the main contributions and innovations of this study are as follows:

1. To overcome the limitations of single models in fully capturing the diverse characteristics of IoT traffic, this study proposes an intrusion detection model based on CNN-BiLSTM-Transformer. The model uses CNN to extract local features, BiLSTM to model temporal dependencies, and Transformer to integrate global relationships.
2. To address the issue of data imbalance, Borderline-SMOTE is employed to oversample minority classes. In addition, Isolation Forest and Local Outlier Factor (LOF) are applied to reduce noise in the dataset.
3. To address the presence of numerous redundant features in the raw datasets, this research employs three strategies—XGBoost, Chi-square (Chi2), and Mutual Information—to effectively select critical features, ensuring that the model focuses on the most discriminative ones.

The rest of this paper is organized as follows: Section 2 presents the proposed intrusion detection methodology. Section 3 details the experimental setup and results analysis. Section 4 concludes the paper and discusses future directions.

## 2. Intrusion Detection Method

### 2.1. Overall Framework of the Intrusion Detection Model

**Figure 1:**
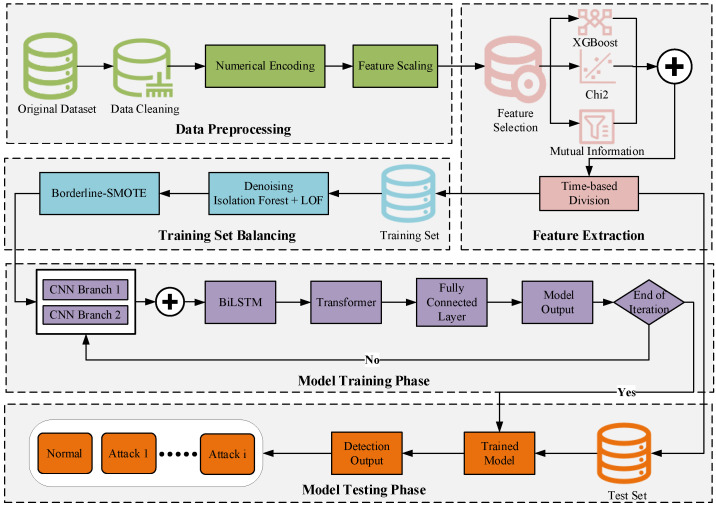
Overall framework diagram.

The overall framework of the proposed CNN-BiLSTM-Transformer-based intrusion detection method is illustrated in Figure 1. The model integrates three strategies—XGBoost, Chi-square (Chi2), and Mutual Information—for key feature selection, and combines Isolation Forest and LOF to eliminate noise and redundant data. Borderline SMOTE is used to address class imbalance. During model building, two parallel CNN branches are employed to extract multi-scale local features. BiLSTM then captures temporal dependencies, and the Transformer module integrates global feature relationships. In this context, BiLSTM is capable of capturing temporal features in network traffic simultaneously from both forward and backward directions. This makes it particularly effective for handling intrusion detection data with significant time dependencies. In contrast to traditional one-way LSTM, BiLSTM provides more comprehensive contextual information, enhancing the model’s ability to identify complex attack behaviors. Given that datasets such as CIC-IDS2017 inherently possess time sequence characteristics, incorporating BiLSTM allows for a more effective modeling of the dynamic evolution process of attack behaviors. Compared to existing studies, this paper introduces several strategies during the feature engineering phase to enhance the quality of feature representation. Additionally, it constructs a hybrid deep learning architecture that integrates local feature extraction, temporal modeling, and global relationship learning. This improves the model’s intrusion detection capabilities and generalization performance in complex network environments. Notably, to ensure fair model evaluation and prevent data leakage issues—especially considering the clear time series nature of the CIC-IDS2017 dataset—the paper adopts a time-order-based division strategy during the data partitioning process. Traffic data from specific dates are assigned to the training set, while data from other dates are reserved for the test set. This ensures complete separation between the training and test sets in the time dimension, avoiding any overlap. By preventing data from the same time period from appearing in both sets simultaneously, this approach ensures the reliability of the experimental results.

### 2.2. Introduction to the Dataset

The CIC-IDS2017 dataset (Canadian Institute for Cybersecurity Intrusion Detection System, 2017) records network traffic between July 2017 and July 2018, encompassing both normal and attack data generated in real-world network environments. The dataset is large-scale, containing 2,830,743 network traffic records, including 2,273,097 normal and 557,646 attack instances [17]. Due to its origin in real-world network environments and the similarity of its attack patterns to IoT threats, CIC-IDS2017 is widely used for IoT intrusion detection research. Table 1 details the data distribution.

The BoT-IoT (Botnet of Things—Internet of Things) dataset was released by the University of New South Wales (UNSW) in Australia in 2018. It is a high-quality dataset for intrusion detection, specifically designed to cover various types of attack scenarios in Internet of Things environments. It includes many common types of network attacks, such as DoS, DDoS, port scanning, and data theft. It also contains normal traffic, making it both representative and challenging [18].

### 2.3. Data Preprocessing

Data preprocessing plays a vital role in enhancing model performance. First, data cleaning replaces missing and infinite values with zeros, and removes outliers. Features such as “FlowPackets/s” are encoded to ensure consistency. Benign traffic is labeled as 0, and attack traffic is labeled as 1. Finally, normalization eliminates scale differences and improves model convergence and learning efficiency. This method unifies feature ranges and optimizes learning across multi-dimensional features [19]. The specific calculation formula is shown in Equation (1).

(1) $$Norm(x)=\frac{x-min(X)}{max(X)-min(X)}$$

where Norm(*x*) is the normalized value of feature *x*, and *min*(*X*) and *max*(*X*) are the minimum and maximum values of the feature in the dataset [20].

### 2.4. Feature Selection

Feature selection plays a crucial role in intrusion detection systems. As dataset dimensionality increases, redundant features and noise can increase computational complexity, lengthen training time, and increase the risk of overfitting [13]. Effective feature selection improves model performance while reducing resource consumption. This study combines XGBoost, Chi-square (Chi2) and Mutual Information for feature screening.

XGBoost, a gradient-boosted decision tree model, evaluates feature importance based on split contribution scores or gains and excels at capturing non-linear relationships in complex datasets [21]. The specific calculation formula is shown in Equation (2).

(2) $$Importance(f)=\sum_{t\in T(f)}\frac{g_{t}}{h_{t}}$$

where $g_{t}$ and $h_{t}$ are the gradient and Hessian matrix of the node in tree *t*. Features are ranked and selected based on importance values.

Chi-square (Chi2) measures the association between features and target variables by evaluating the deviations between observed and expected frequencies. Its core principle is to assess the importance of features by calculating the degree of deviation between actual observed values and theoretical expectations [22]. The specific calculation formula is shown in Equation (3).

(3) $$\chi^{2}=\sum \frac{{\left(O_{i}-E_{i}\right)}^{2}}{E_{i}}$$

where $O_{i}$ and $E_{i}$ are observed and expected frequencies. Chi2 efficiently identifies features significantly correlated with the target.

Mutual Information quantifies the dependency between features and target variables, capturing nonlinear relationships [23]. The specific calculation formula is shown in Equation (4).

(4) $$I(A;B)=\sum_{a\in A}\sum_{b\in B}P(a,b)log\frac{P(a,b)}{P(a)P(b)}$$

where *P*(*a*, *b*) is the joint probability, and *P*(*a*) and *P*(*b*) are the marginal probabilities. Mutual Information is not only applicable to discrete data but can also handle continuous features, thus having a broader range of applications.

**Figure 2:**
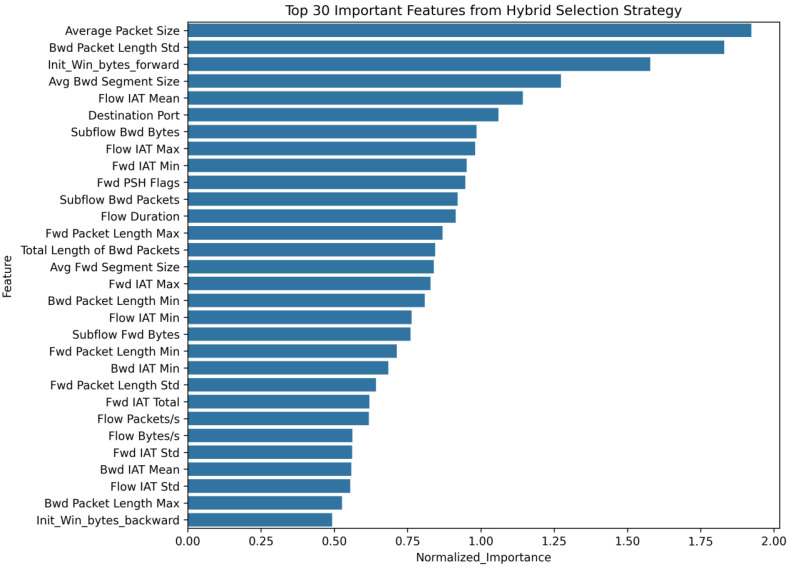
Sorting chart of the top 30 important features.

This study employs a multi-stage feature selection strategy to optimize the feature selection process. First, XGBoost is used to perform a preliminary evaluation of feature importance. Second, Chi-square (Chi2) and Mutual Information methods are combined to conduct secondary screening of features from the perspectives of statistical relevance and information dependency. Finally, the results of the three methods are integrated to determine the optimal feature subset. This hybrid feature selection strategy fully accounts for nonlinear relationships among features, classification relevance, and information dependency. By reducing computational complexity and overfitting risks, it effectively enhances the model’s ability to capture temporal and global features. Experiments on the CIC-IDS2017 dataset confirm that 52 features are ultimately selected as model inputs after hybrid method screening. Figure 2 illustrates the distribution of the top 30 important features.

### 2.5. Class Imbalance Handling

In intrusion detection research based on the CIC-IDS2017 dataset, the class distribution imbalance is particularly prominent. In this dataset, samples of benign traffic significantly outnumber samples of attack traffic, and this imbalance often biases model predictions towards the normal class [24]. To address this issue, this paper employs the Borderline-SMOTE method for oversampling and combines it with Isolation Forest and Local Outlier Factor (LOF) techniques for noise filtering.

Borderline-SMOTE (Borderline Synthetic Minority Over-sampling Technique) is an advanced version of SMOTE specifically designed to oversample minority class samples near decision boundaries. Compared to standard SMOTE, this method focuses more on generating samples in classification boundary regions. By calculating distances between samples and using interpolation to synthesize new ones, it significantly improves the identification of minority class instances and effectively mitigates class imbalance [25].

To deal with noise, this study integrates two anomaly detection techniques: Isolation Forest and LOF. Isolation Forest identifies outliers by randomly partitioning the feature space, where anomalous samples are isolated in fewer partitioning steps [26]. The LOF algorithm detects anomalies by computing local density discrepancies, effectively identifying low-density data points [27]. Isolation Forest excels at global anomaly detection, while LOF specializes in local outlier detection. Their combination allows for a more comprehensive removal of noise and redundant information from the dataset.

### 2.6. CNN-BiLSTM-Transformer Model

In intrusion detection tasks, CNN can effectively extract key features from data and is particularly suitable for processing spatially structured data [28]. However, CNN has limitations in capturing global dependencies in sequential data, especially for time-dependent sequences. To overcome this limitation, this paper’s approach incorporates BiLSTM, which extracts temporal features in both the forward and backward directions, making it particularly adept at capturing temporal dependencies in data [29]. Although BiLSTM performs well in processing sequential data, it faces high computational complexity when handling complex long-term dependencies and exhibits lower efficiency in long-sequence processing. To further enhance model performance, this paper incorporates a Transformer module. The Transformer leverages its multi-head attention mechanism to capture global dependencies and addresses BiLSTM’s bottleneck in long-sequence processing by enabling parallel computation [30]. Therefore, this work combines CNN, BiLSTM, and Transformer modules to design an optimized CNN-BiLSTM-Transformer model, which better handles complex features and long-sequence dependencies in intrusion detection.

**Figure 3:**
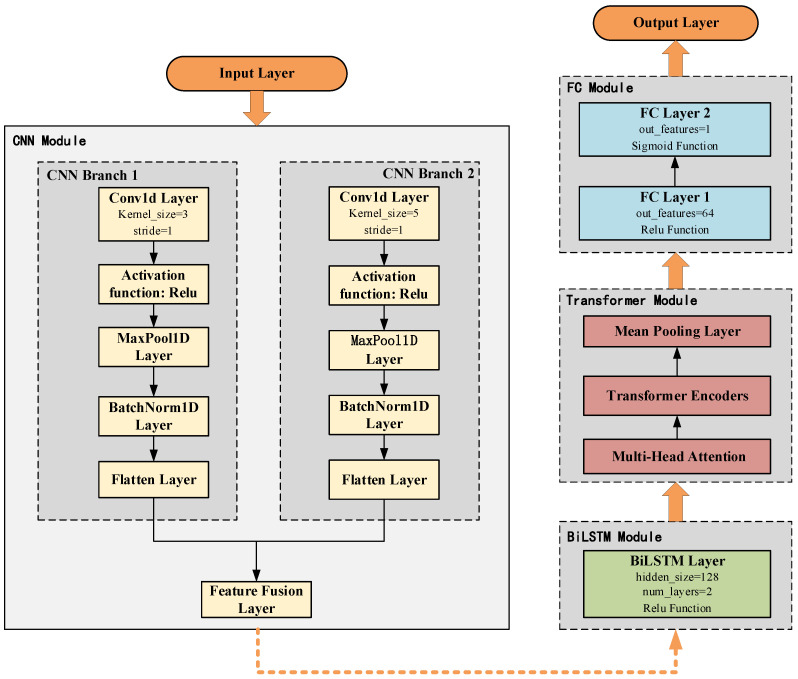
Model structure.

The model architecture is illustrated in Figure 3. Input data is fed into two parallel CNN branches for feature extraction—one using a small convolutional kernel and the other a large one. After merging features from both branches, BiLSTM is employed to extract temporal sequence features. The output of BiLSTM undergoes global information modeling via the multi-head attention mechanism and Transformer encoder, and final classification is performed through a fully connected layer.

CNN possesses the capability to automatically extract features from data, effectively capturing spatial patterns within the data. The core operation of CNN is convolution, which performs element-wise multiplication and summation on input data within sliding windows [31]. In this model, two convolutional branches are employed: one uses a smaller convolutional kernel (size 3), and the other uses a larger kernel (size 5). Each branch processes the input through the following steps:

1. 1D convolutional layer (Conv1d) for feature extraction.
2. Activation function for non-linear transformation.
3. Max pooling (MaxPool1d) to reduce matrix dimensions.
4. Batch normalization (BatchNorm1d) to accelerate training and stabilize learning.
5. Flatten operation to convert features into a 1D vector [32].

The outputs from both branches are then merged to form the final feature representation. The mathematical formula for the convolution operation is expressed as follows:

(5) $$y(t)=(x*w)(t)=\sum_{i=0}^{k-1}x(t+i)w(i)$$

where *x*(*t*) represents the input signal, *w*(*i*) denotes the convolutional kernel, *y*(*t*) is the output signal, and k is the size of the convolutional kernel.

Although CNN effectively extracts local features, its feedforward architecture—where outputs from neurons are only propagated to subsequent layers—fails to adequately analyze temporal correlations in data. This limitation prevents CNN from fully capturing temporal features in intrusion data [33]. To address this, the model incorporates a Bidirectional Long Short-Term Memory network (BiLSTM). BiLSTM employs two LSTM units to independently process input sequences in both forward and backward directions, enabling comprehensive capture of temporal dependencies.

The LSTM unit alleviates the long-term dependency problem in traditional RNNs by introducing input gates, forget gates, and output gates. These gating mechanisms allow LSTM to efficiently propagate critical information across long sequences while mitigating gradient-vanishing issues [34]. The mathematical formulas for each gate are defined in Equations (6)–(10). In BiLSTM, the forward and backward outputs are concatenated to form a richer feature representation, which is then fed as input to subsequent layers [35].

(6) $$i_{t}=\sigma (W_{i}[h_{t-1},x_{t}]+b_{i})$$

The input gate determines the influence of the current input information on the current state. In the formula, σ represents the sigmoid activation function, and the output value *i_t_* lies within the range [0, 1], reflecting the significance of the current input data. Here, *W_i_* denotes the weight matrix for the input gate, *h_t_*_−1_ represents the hidden state from the previous timestep, and *x_t_* is the input data at the current timestep [36].

(7) $$f_{t}=\sigma (W_{f}[h_{t-1},x_{t}]+b_{f})$$

The forget gate controls the retention of memory information from the previous timestep at the current timestep. In the formula, *f_t_* is the output of the forget gate, representing the proportion of historical information retained at the current timestep [37].

(8) $$o_{t}=\sigma (W_{o}[h_{t-1},x_{t}]+b_{o})$$

The output gate determines the contribution of the current memory information to the output. *o_t_* is the value of the output gate, governing the update degree of the final hidden state *h_t_* [38].

(9) $$c_{t}=f_{t}c_{t-1}+i_{t}\tanh\left(W_{c}[h_{t-1},x_{t}]+b_{c}\right)$$

The current memory cell *c_t_* is formed by the weighted combination of the previous memory cell *c_t_*_−1_ and the current input data. The forget gate *f_t_* controls how much of the previous memory is retained, while the input gate *i_t_* governs the contribution of the current input to the memory [14].

(10) $$h_{t}=o_{t}\tanh\left(c_{t}\right)$$

The hidden state *h_t_* is regulated by the output gate *o_t_* and integrated with the processed data from the memory cell *c_t_*. The final hidden state *h_t_* encapsulates information from the current timestep and long-term dependencies from prior timesteps [39].

Although BiLSTM performs well in processing sequential data, it suffers from high computational complexity and low efficiency when handling long sequences. To enhance the model’s capability for long-sequence processing, this paper introduces the Transformer module. The Transformer module integrates global relationships through the multi-head attention mechanism and Transformer encoder [40]. Its computational core is the Self-Attention mechanism, whose formula is defined in Equation (11).

(11) $$Attention(Q,K,V)=\mathrm{softmax}\left(\frac{QK^{T}}{\sqrt{d_{k}}}\right)V$$

In this formula, *Q* is the query matrix, *K* is the key matrix, *V* is the value matrix, and dk is the dimension of the keys. The query matrix *Q* and key matrix *K* undergo matrix multiplication to compute similarity scores, which are then scaled and normalized to obtain attention weights. Finally, the attention weights are multiplied with the value matrix *V* to generate a weighted output [15].

The multi-head attention mechanism computes multiple attention heads in parallel. The input Query, Key, and Value are linearly transformed into formats suitable for scaled dot-product attention. The scaled dot-product attention is then applied to compute attention weights. The outputs of multiple heads are concatenated and passed through another linear transformation to produce the final output [16]. In the Transformer, multiple encoder layers are stacked using multi-head attention mechanisms and feed-forward neural networks (FFN). The output of each encoder layer is computed using Equation (12).

(12) Output=MultHeadAttention(Q,K,V)+FFN⁡(MultiHeadAttention)

FFN (Feed-Forward Neural Network) typically consists of two linear transformations and a ReLU activation function. This network is applied to further analyze the outputs of multiple attention heads, aiding in capturing more complex patterns. By stacking multiple encoder layers and leveraging the multi-head attention mechanism to capture global relationships within sequences, the Transformer achieves efficient information extraction on long-sequence data [41].

## 3. Experimental Results and Analysis

### 3.1. Experimental Environment and Parameter Settings

The software environment versions, hardware configurations, and model parameter settings used in this experiment are detailed in Table 2.

### 3.2. Experimental Results Analysis

**Figure 4:**
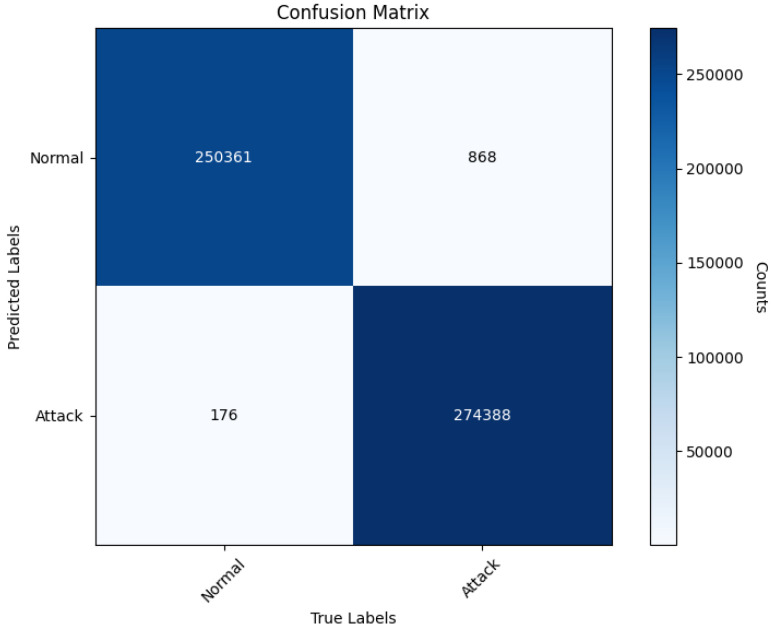
Confusion Matrix.

In this section, the model was evaluated and analyzed using the CIC-IDS2017 dataset. The confusion matrix for the experiment is presented in Figure 4. The findings indicate that the model successfully predicts the majority of both normal and attack traffic instances. However, 868 instances of normal traffic were incorrectly classified as attacks (false positives), and 176 attack instances were misclassified as normal traffic (false negatives). Upon examining the distribution of classification errors, it is evident that the number of false positives slightly exceeds that of false negatives. This may be attributed to certain normal traffic patterns being closely aligned with attack patterns within the feature space. Particularly at the boundaries where feature overlap occurs, these instances are prone to misclassification. Although the Borderline-SMOTE technique alleviated the issue of class imbalance, real-world network environments often exhibit high variability and unpredictability in attack behaviors. This could result in some abnormal sample features resembling normal behaviors, thereby causing incorrect predictions. Based on the confusion matrix, the model achieves an accuracy of 99.80%, a precision of 99.69%, a recall of 99.94%, an F1-score of 99.81%, an AUC of 0.9987, a false positive rate (FPR) of only 0.0034, and a detection rate (DR) of 99.94%. These metrics suggest that the model performs exceptionally well in intrusion detection tasks. It effectively distinguishes between normal and attack traffic while maintaining low rates of false alarms and missed detections, thus showcasing its robustness and dependability.

**Figure 5:**
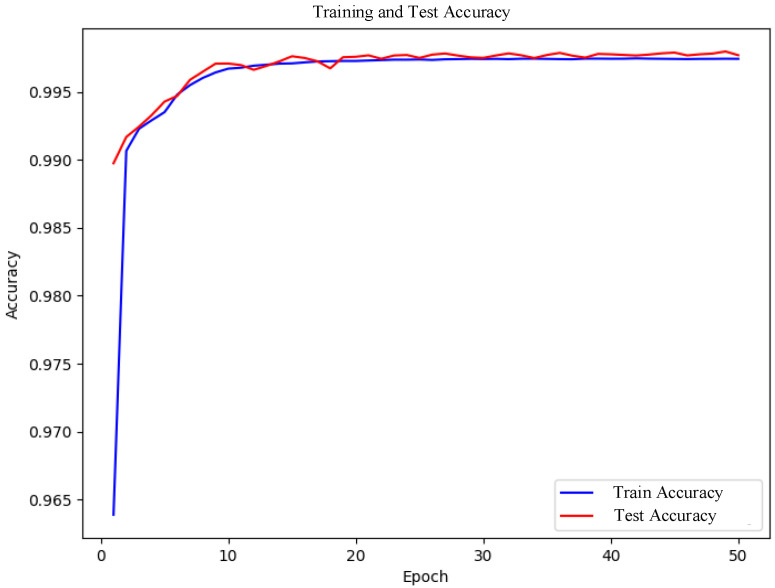
Model accuracy graph.

Figure 5 displays the accuracy curves during model training. As shown in the figure, both training accuracy and test accuracy rise rapidly and stabilize around the 10th epoch. The close alignment between test accuracy and training accuracy indicates no overfitting. The rapid improvement in training accuracy demonstrates the model’s ability to effectively learn features from training data. The stability of test accuracy and its proximity to training accuracy further confirm the model’s strong generalization capability on unseen data. This consistently high accuracy underscores the superior classification performance of the proposed CNN-BiLSTM-Transformer hybrid model in IoT intrusion detection tasks. The integration of Borderline-SMOTE effectively mitigates class imbalance, enabling the model to better learn features of the minority class, thereby enhancing overall detection efficacy.

**Figure 6:**
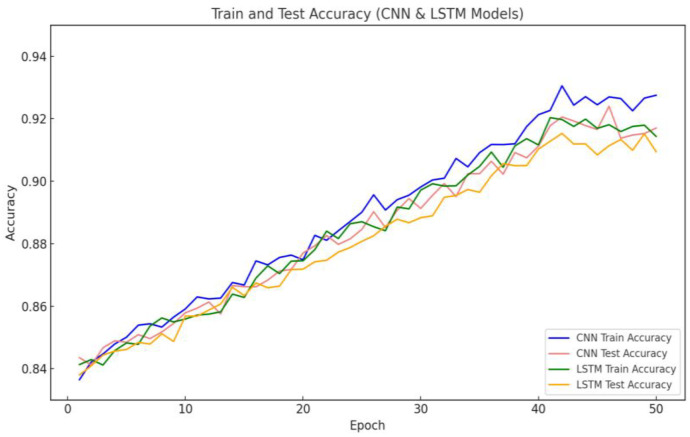
Training and testing accuracy curves of baseline model.

Nevertheless, the model achieved an accuracy of approximately 96.5% almost immediately at the onset of training. This could indicate that the dataset is relatively easy to learn or that the model possesses a robust capacity for feature extraction. To further investigate this and rule out the possibility of overfitting or data leakage, this study plotted the training and testing accuracy curves of the baseline models (CNN and LSTM), as shown in Figure 6. The results show that both models exhibited a markedly slower accuracy increase in the initial training phase. Ultimately, they attained training accuracies of roughly 92.5% and 91.9%, respectively, which were lower than that of the proposed model. This demonstrates that the model presented here has clear advantages in terms of feature representation and learning efficiency. Hence, this phenomenon is not attributable to data issues but rather to the efficacy of the model architecture itself. Additionally, to ensure the reproducibility of the training process and fair comparison of model performances, all deep learning models in this study were trained under identical data splits, preprocessing procedures, and training configurations, including the same learning rates, optimizers, batch sizes, and other parameter settings. Regarding parameter initialization, the convolutional layers and fully connected layers utilized the Kaiming Normal initialization strategy based on the He normal distribution. Meanwhile, the LSTM and Transformer modules employed the default Xavier initialization method available in PyTorch. Although parameter initialization strategies contribute to improving the stability and convergence speed of model training to some extent, the phenomenon of achieving high accuracy at an early stage of training is primarily attributed to the synergistic effect of model architecture design and data preprocessing strategies. On the one hand, the CNN-BiLSTM-Transformer architecture enables efficient extraction of local features, modeling of temporal dependencies, and integration of global information during the initial training phase, thereby accelerating the capture of effective feature representations. On the other hand, feature selection and noise sample elimination enhance data quality, allowing the model to learn more representative feature patterns from the beginning of training. Additionally, the use of Borderline-SMOTE alleviates class imbalance, enabling the model to identify minority class features early on, which further improves overall detection performance.

To further verify the robustness and generalization capability of the proposed model, and to rule out the possibility that the high performance was caused by random initialization, we conducted five independent training experiments under identical data splitting, preprocessing procedures, and training parameter settings. The results show that the average training accuracy was 99.42% with a standard deviation of 0.46%, and the average testing accuracy was 99.34% with a standard deviation of 0.37%. The slightly lower test deviation suggests that the model’s generalization is less sensitive to initialization variations. Although minor fluctuations occurred across runs, they are mainly due to the inherent randomness in model initialization and occasional misclassification of rare attack samples. Nevertheless, the performance remained reliably high (within ±0.5% deviation), demonstrating that the proposed CNN-BiLSTM-Transformer hybrid architecture exhibits strong stability and reproducibility in IoT intrusion detection tasks. Figure 5 shows the training and testing accuracy curves for the experimental group with the best performance. Therefore, the high accuracy achieved in the early stages of training is not incidental or merely the result of initialization, but rather the outcome of multiple coordinated improvements.

**Figure 7:**
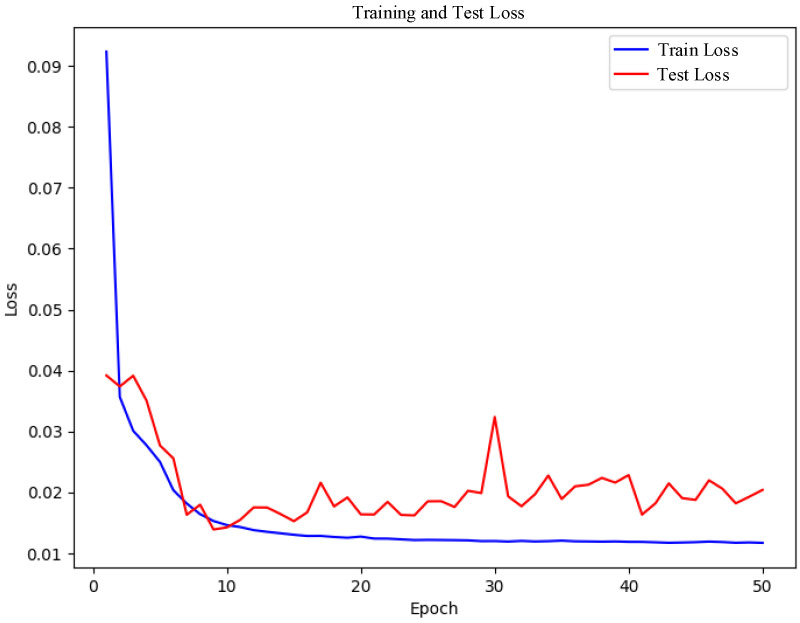
Model loss graph.

Figure 7 illustrates the loss curves observed during the model’s training phase. As depicted in the figure, the training loss decreases rapidly and stabilizes after approximately the tenth epoch. Initially, the test loss exhibits slight fluctuations but subsequently declines gradually, eventually settling at a low value. The rapid reduction in training loss indicates that the model effectively minimizes the loss function and optimizes its parameters. The gradual decrease and eventual stabilization of the test loss at a low level further confirm the model’s strong performance on the test dataset. This consistently low loss demonstrates that the model continues to improve throughout training and exhibits robust generalization capabilities, adapting effectively to the test data.

By employing a combination of XGBoost, Chi-square tests, and mutual information for feature selection, the model successfully eliminates redundant features, enabling it to concentrate on learning the most relevant ones. This process not only reduces the loss further but also enhances the model’s generalization ability. Additionally, the application of Isolation Forest and LOF techniques effectively identifies and removes noise and outliers from the data, thereby minimizing interference during training and resulting in smoother loss curves and faster convergence.

### 3.3. Comparative Experiments

**Figure 8:**
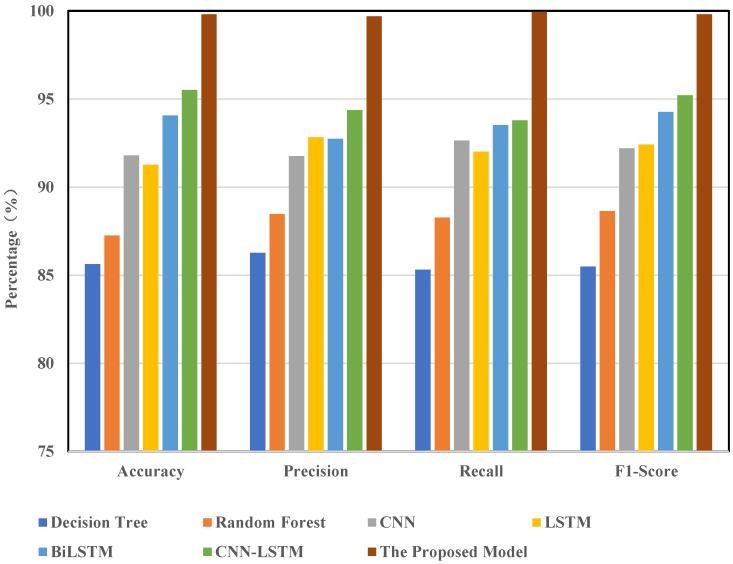
Comparison of different model detection results.

This study demonstrates the practical applicability of the proposed model via comparative experiments. In this section, the model was evaluated and analyzed using the CIC-IDS2017 dataset. We selected several mainstream models for comparison, including decision trees, random forests, CNN, LSTM, BiLSTM, CNN-LSTM, and CNN-BiLSTM. All models were trained and tested on the same dataset with an identical preprocessing pipeline (which included feature selection, oversampling, and noise reduction). The results of these comparisons are shown in Figure 8.

The findings indicate that the model presented in this paper performs exceptionally well across all evaluation metrics. In terms of accuracy (ACC), our model achieves 99.80%, compared to 91.80% for CNN and 91.27% for LSTM. For precision, recall, and F1-score—three critical metrics—our model attains values of 99.69%, 99.94%, and 99.81%, respectively. Its overall performance significantly surpasses that of other baseline models. Notably, in terms of recall, our model outperforms CNN-LSTM by approximately 3 percentage points. These experimental results clearly demonstrate that our model exhibits superior detection accuracy and stability in intrusion detection tasks, surpassing existing mainstream approaches.

The primary reason for this performance advantage lies in the structural design of the proposed CNN-BiLSTM-Transformer model, which integrates three key capabilities: spatial feature extraction, temporal dependency modeling, and global relationship capture. This effectively addresses the limitations of traditional single models, which struggle to recognize complex attack patterns. While conventional machine learning methods such as decision trees and random forests possess some generalization ability, they fail to model temporal relationships and deep features within traffic data, making it challenging for them to handle intricate attack behaviors. Although CNN can extract local spatial features, it overlooks temporal dependencies. Similarly, while LSTM is capable of modeling temporal information, it struggles with capturing global dependencies and long-range correlations. In contrast, our model first extracts spatial features using CNN, then models bidirectional temporal dependencies through BiLSTM, and finally captures global associations via the Transformer module. This significantly enhances the model’s capacity to understand and distinguish complex attack patterns. Additionally, optimization techniques such as feature selection, oversampling, and denoising further enhance data quality and model robustness, resulting in a substantial improvement in overall performance.

To evaluate the effectiveness of the proposed CNN-BiLSTM-Transformer hybrid model for IoT intrusion detection, this paper selects several mainstream intrusion detection methods—such as BiGAN, CNN + BiLSTM + SMOTE, BaysCNN + PCA, TranBiLSTM + ResNet, Deep Autoencoder, Hybrid RNN, and EvoBMF + BiLSTM + MHA—as baselines for comparison. Table 3 shows how these different intrusion detection methods perform in terms of performance indicators.

The experimental results show that the accuracy of the proposed model is 99.80%. This is higher than the CNN + BiLSTM + C5.0 model in [6] and the BaysCNN + PCA model in [9]. In contrast, the accuracies of the EvoBMF + BiLSTM + MHA model in [10] and the BiGAN model in [42] are 90.31% and 82.30%, respectively. There is a significant performance gap compared to the proposed model. Also, the model in this paper is 1.73 percentage points better than the 1D Conv-BiLSTM + Attention model in [7]. This indicates that the proposed model has a stronger capability to detect various types of attacks. In terms of precision, the proposed model achieves 99.69%, slightly higher than the BaysCNN + PCA model (97.69%). However, the BaysCNN + PCA model performs worse than the proposed model in terms of recall and F1-score. This indicates its limitations in achieving comprehensive detection. By combining the Transformer and CNN-BiLSTM structure and using the Borderline-SMOTE method to address data imbalance, the accuracy and stability of the proposed model have been further improved.

Recall is an important metric for evaluating the model’s ability to detect attacks. The proposed model achieves a recall rate of 99.94%, which is significantly higher than that of other methods. Additionally, by using Isolation Forest and LOF to remove noise and redundant information, the model’s ability to detect minority class attacks has been enhanced. In terms of the F1-score, the proposed model achieves 99.81%, the highest among all methods considered. In contrast, the 1D Conv-BiLSTM + Attention model in [7] is 98.61%, the CNN + BiLSTM + SMOTE model in [8] is 97.75%, and the BiGAN model in [42] is only 76.40%. The significant improvement in F1-score demonstrates that the proposed model achieves a better balance between precision and recall. It can deal with all kinds of attack threats in the IoT environment more completely.

### 3.4. Ablation Experiments

In this section, we conducted ablation experiments to evaluate how each module affects the model’s performance. We removed key components—such as oversampling techniques, feature selection, and the Transformer module—to observe their impact on model performance. The experiments were based on the CIC-IDS2017 dataset and used Borderline-SMOTE oversampling, feature selection strategies, and Isolation Forest and LOF denoising methods for data preprocessing. The results of the ablation experiments are shown in Table 4.

The experimental results show the following:

(1) After removing the oversampling technique, the model performance decreased significantly, with accuracy dropping to 96.76%, precision dropping to 96.56%, recall dropping to 94.14%, and F1-score dropping to 96.87%. This shows that Borderline-SMOTE effectively mitigates class imbalance and improves the model’s recall and accuracy. The oversampling technique enhances the detection of minority class attacks by generating synthetic samples, enabling the model to learn their characteristics more effectively.

(2) After removing feature selection, accuracy dropped to 97.46%, precision to 95.29%, recall to 94.98%, and F1-score to 96.13%. Feature selection reduces redundant information; removing it causes the model to rely on irrelevant features, leading to performance degradation. By integrating XGBoost, Chi-square test and mutual information for feature selection, the model focuses on learning critical features, thereby improving detection efficiency and accuracy.

(3) After removing the Transformer module, accuracy dropped to 95.92%, precision to 94.17%, recall to 93.20%, and F1-score to 95.41%. This highlights the central role of the Transformer module in global information modelling. The module captures long-range dependencies in the data through its self-attention mechanism, which enhances the model’s ability to recognize complex attack patterns.

Through ablation experiments, this study validates the importance of each module in the model. The results confirm that Borderline-SMOTE, feature selection strategies, and the Transformer module all contribute significantly to model performance. The synergistic effects of these modules ensure that the proposed CNN-BiLSTM-Transformer hybrid model achieves outstanding performance in IoT intrusion detection, delivering high accuracy, precision, recall, and F1-score.

### 3.5. Analysis of Experimental Results on the BoT-IoT Dataset

**Figure 9:**
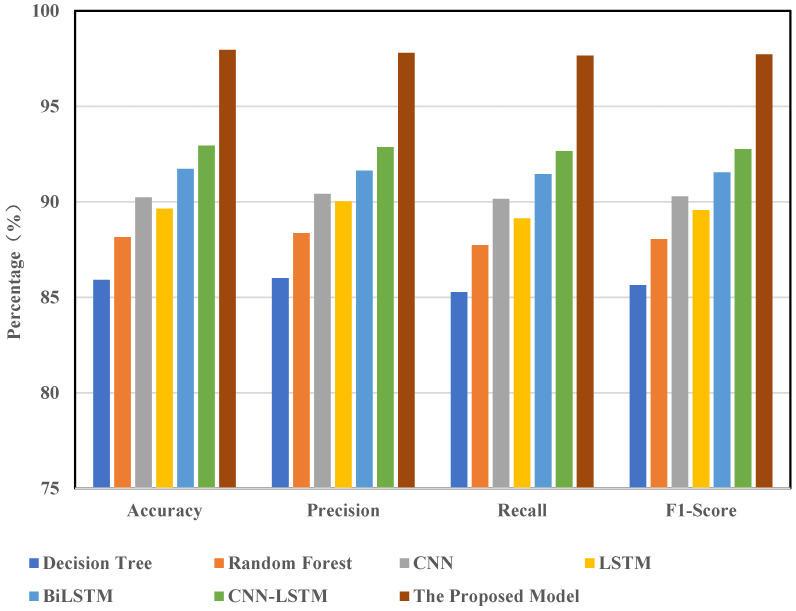
Comparison of detection results of different models in BoT-IoT datasets.

To further demonstrate the effectiveness and generalizability of the proposed CNN-BiLSTM-Transformer model in Internet of Things (IoT) scenarios, this study conducted comparative experiments using the BoT-IoT dataset. The experiments compared the proposed model with mainstream models such as decision trees, random forests, CNN, LSTM, BiLSTM, CNN-LSTM, and CNN-BiLSTM. All models were trained and evaluated using the same dataset and preprocessing steps, which included feature selection, oversampling, and noise reduction. The experimental results are illustrated in Figure 9.

The results indicate that the proposed model outperforms all other models across all evaluation metrics. It achieved an accuracy of 97.95%, precision of 97.80%, recall of 97.65%, and F1-score of 97.72%. Compared to its performance on the CIC-IDS2017 dataset, the model shows a slight decrease in effectiveness. This is mainly due to the increased complexity of attack types and the more imbalanced sample distribution in the BoT-IoT dataset, especially the challenge of detecting minority attack classes. Among the comparison models, the best-performing one, CNN-BiLSTM, achieved an accuracy of 92.94% and an F1-score of 92.76%, which is still approximately 5 percentage points lower than the proposed model. Other models, such as BiLSTM (F1-score of 91.54%), CNN (90.28%), LSTM (89.57%), random forest (88.05%), and decision tree (85.64%), performed significantly worse. These results highlight the superiority of the proposed model in handling complex attack patterns and addressing data imbalance issues in IoT environments.

Analyzing the differences in model performance reveals the limitations of traditional machine learning methods in IoT settings. Decision trees struggle with temporal information and are prone to overfitting minority classes. Although random forests are effective at resisting noise, they fail to capture sequence dependencies. CNN excels at extracting local spatial features but lacks the ability to model long-term temporal relationships. LSTM can handle time series data but faces challenges in spatial feature extraction and training stability. BiLSTM improves temporal modeling but still struggles with capturing global dependencies. While CNN-LSTM and CNN-BiLSTM integrate spatial and temporal modeling capabilities, they remain limited in terms of global perception. In contrast, the proposed CNN-BiLSTM-Transformer model employs an hierarchical structure to sequentially perform spatial feature extraction (via CNN), bidirectional temporal modeling (via BiLSTM), and global dependency capture (via Transformer). This approach significantly enhances the model’s ability to recognize complex attack behaviors. Additionally, by incorporating strategies such as feature selection, oversampling, and noise removal, the model becomes more robust, leading to improved overall performance.

## 4. Conclusions

In this study, a hybrid model combining CNN, BiLSTM, and Transformer is introduced to address the challenges faced by traditional intrusion detection systems in handling complex attack types, data imbalance, and feature extraction difficulties within the Internet of Things (IoT) environment. To tackle the issue of data imbalance, the Borderline-SMOTE technique is integrated into the intrusion detection framework. Feature selection is performed using XGBoost, Chi-square tests, and mutual information methods to enhance the model’s detection capabilities. Additionally, Isolation Forest and Local Outlier Factor (LOF) algorithms are employed to eliminate noise and redundant information from the dataset. Experimental results demonstrate that this approach achieves accuracies of 99.80% and 97.95% on the CIC-IDS2017 and BoT-IoT datasets, respectively. These outcomes surpass those of conventional intrusion detection techniques, confirming the effectiveness and robustness of the proposed model in identifying abnormal traffic patterns in IoT settings.

Despite demonstrating promising detection performance, the proposed method has certain limitations: (1) The deep neural network architecture, while delivering high accuracy, requires significant computational resources, which may pose challenges when deploying on resource-constrained IoT devices. (2) The experimental validation primarily relies on offline datasets; further evaluation is necessary to assess its real-time traffic detection capabilities. To overcome these limitations, future research will explore the following key areas: (i) Investigating lightweight strategies such as model compression, parameter sharing, and knowledge distillation to maintain high detection accuracy while enhancing adaptability and resource efficiency for deployment on edge devices. (ii) Developing an end-to-end real-time intrusion detection system prototype to evaluate the model’s performance in actual IoT environments and achieve sub-millisecond response times for attack detection. (iii) Extending this methodology to specialized domains like the Industrial Internet of Things (IIoT) and Vehicular Ad-hoc Networks (V2X), tailoring it to meet the unique security requirements of these fields.

## Figures and Tables

**Table 1:** Description of the distribution of the CIC-IDS2017 dataset.

ID	Data Category	Quantity	Percentage
1	BENIGN	2,273,097	80.3000%
2	DoS Hulk	231,073	8.1629%
3	PortScan	158,930	5.6144%
4	DDoS	128,027	4.5227%
5	DoS GoldenEye	10,293	0.3636%
6	FTP-Patator	7938	0.2804%
7	SSH-Patator	5897	0.2093%
8	DoS slowloris	5796	0.2047%
9	DoS Slowhttptest	5499	0.1942%
10	Bot	1966	0.0694%
11	Web Attack-Brute Force	1507	0.0532%
12	Web Attack-XSS	652	0.0230%
13	Infiltration	36	0.0012%
14	Web Attack-Sql Injection	21	0.0007%
15	Heartbleed	11	0.0004%

**Table 2:** Configuration of experimental environment and model parameters.

Configuration Item/Parameter	Value	Selection Justification
CPU Version	Intel i5-10300H (Intel Corporation, Santa Clara, CA, USA)	Meets computational requirements
GPU Version	NVIDIA GTX 1650 Ti (NVIDIA Corporation, Santa Clara, CA, USA)	4GB VRAM sufficient for model training
Operating System	Windows 10 (Microsoft Corporation, Redmond, WA, USA)	Stable platform with framework compatibility
Python Version	Python 3.10.14 (Python Software Foundation, Wilmington, DE, USA)	PyTorch-recommended version
Deep Learning Framework	PyTorch 1.12.1 (Meta Platforms, Inc., Menlo Park, CA, USA)	Base framework for this study
Training Epochs	50	Early stopping (validation loss, patience = 5)
Learning Rate	0.001	Optimal via grid search (0.1, 0.01, 0.001, 0.0001)
Batch Size	64	Memory-efficiency balance (tested 32/64/128)
Optimizer	Adam	Default parameters (β_1_ = 0.9, β_2_ = 0.999)
Loss Function	Binary Cross-Entropy Loss	Standard for binary classification tasks

**Table 3:** Comparison of different intrusion detection methods.

Reference	Method	Accuracy	Precision	Recall	F1-Score
[6]	CNN + BiLSTM + C5.0	95.50%	95.32%	95.13%	95.22%
[7]	1D Conv-BiLSTM + Attention	98.07%	98.81%	98.42%	98.61%
[8]	CNN + BiLSTM + SMOTE	97.70%	97.80%	97.70%	97.75%
[9]	BaysCNN + PCA	99.66%	97.69%	97.69%	97.69%
[10]	EvoBMF + BiLSTM + MHA	90.31%	93.28%	88.24%	90.69%
[11]	Multi-scale Transformer	99.25%	99.07%	99.02%	99.04%
[16]	TranBiLSTM + ResNet	99.15%	99.15%	99.14%	99.14%
[42]	BiGAN	82.30%	76.50%	76.30%	76.40%
[43]	Deep autoencoder	98.23%	98.17%	98.29%	98.23%
[44]	Hybrid RNN	82.91%	86.78%	82.91%	84.79%
The Proposed Model	CNN + BiLSTM + Transformer	99.80%	99.69%	99.49%	99.81%

**Table 4:** Results of ablation experiments.

	Accuracy	Precision	Recall	F1-Score
The Proposed Model	99.80%	99.69%	99.94%	99.81%
Without Oversampling	96.76%	96.56%	94.14%	96.87%
Without Feature Selection	97.46%	95.29%	94.98%	96.13%
Without the Transformer Module	95.92%	94.17%	93.20%	95.41%

## Data Availability

The original data presented in the study are openly available in CIC-IDS2017 at https://www.unb.ca/cic/datasets/ids-2017.html (accessed on 1 October 2024).
